# Efficacy of Atezolizumab in Subsequent Lines of Therapy for NSCLC Patients: Insights from Real-World Data

**DOI:** 10.3390/cancers16213696

**Published:** 2024-11-01

**Authors:** Milica Kontić, Filip Marković, Nikola Nikolić, Natalija Samardžić, Goran Stojanović, Petar Simurdić, Svetlana Petkov, Daliborka Bursać, Bojan Zarić, Mihailo Stjepanović

**Affiliations:** 1 Clinic for Pulmonology, University Clinical Centre of Serbia, 11000 Belgrade, Serbia; milica.kontic@med.bg.ac.rs (M.K.); filip.markovic@kcs.ac.rs (F.M.); nikola.nikolic@kcs.ac.rs (N.N.); natalija.samardzic@kcs.ac.rs (N.S.); 2 Faculty of Medicine, University of Belgrade, 11000 Belgrade, Serbia; 3 Institute for Pulmonary Diseases of Vojvodina, 21204 Sremska Kamenica, Serbia; goran.stojanovic@institut.rs (G.S.); petar.simurdic@institut.rs (P.S.); svetlana.petkov@institut.rs (S.P.); daliborka.bursac@mf.uns.ac.rs (D.B.); bojan.zaric@mf.uns.ac.rs (B.Z.); 4 Faculty of Pharmacy, Univerity Business Academy in Novi Sad, 21101 Novi Sad, Serbia; 5 Faculty of Medicine, University of Novi Sad, 21000 Novi Sad, Serbia

**Keywords:** atezolizumab, non-small cell lung cancer, immune checkpoint inhibitors, advanced non-small cell lung cancer

## Abstract

Immune checkpoint inhibitors (ICIs) like atezolizumab have improved outcomes in advanced non-small cell lung cancer (NSCLC) patients, especially in the second-line setting after progression on platinum-based chemotherapy. However, access to ICIs remains limited in many developing nations. This study evaluated the efficacy of atezolizumab as a second-line versus later-line treatment for advanced NSCLC patients in Serbia. Methods: This retrospective study involved 147 advanced NSCLC patients treated with atezolizumab following progression on prior platinum-based chemotherapy at two academic centers in Serbia. Data on demographics and clinical, pathological, and molecular characteristics were collected. Median progression-free survival (PFS) and overall survival (OS) were estimated using the Kaplan–Meier method, and multivariable Cox proportional hazards regression identified outcome predictors. Results: The median PFS was 7.13 months, and median OS was 38.6 months. The overall response rate (ORR) was 15%, with a disease control rate (DCR) of 57.9%. No significant PFS differences were observed between patients treated with atezolizumab in the second line versus later lines. Patients with good performance status (ECOG 0–1) had significantly better PFS compared to those with poorer status (12.03 vs. 1.63 months, *p* < 0.0001). Conclusions: Atezolizumab is effective in both second-line and later-line settings for advanced NSCLC, particularly in patients with good performance status. This highlights the importance of patient selection based on performance status, as well as the need for wider access to ICIs in resource-limited regions.

## Simple Summary

This study analyzes real-world outcomes for advanced, non-oncogene addicted non-small cell lung cancer patients treated with atezolizumab monotherapy following platinum-based chemotherapy, based on data from two academic institutions in Serbia. Progression-free survival did not significantly differ between patients receiving atezolizumab as a second, third, or later line of therapy, indicating consistent efficacy across treatment lines. Additionally, the number of prior chemotherapy cycles had no significant impact on progression-free survival, suggesting that a higher prior treatment burden did not compromise atezolizumab effectiveness. Importantly, a good ECOG performance status emerged as the strongest predictor of prolonged progression-free survival, highlighting the importance of patients’ health status at treatment initiation.

## 1. Introduction

Lung cancer remains the leading cause of cancer-related mortality around the world [1]. Non-small cell lung cancer accounts for 80–85% of all lung cancer cases, and a majority of patients are diagnosed in advanced stages, rendering their condition incurable [2,3]. Improvement in 5-year survival rates among advanced NSCLC patients has been observed in the last decade [4,5].

This gradual improvement could be attributable to the advent of immune checkpoint inhibitors (ICIs), combination treatments, and personalized medicine approaches. ICIs have become a cornerstone in the treatment of non-oncogene addicted advanced NSCLC, especially in the first-line setting [6].

However, their approval status still varies by country based on regulatory decisions influenced by clinical trial data, local healthcare policies, and other factors such as limited, or a lack of, insurance coverage. These factors can restrict access to these therapies, contributing to their uneven global availability in the first-line setting [7,8]. In such instances, when ICI-based treatments are unavailable or the patient is not eligible to receive them in the first-line setting, mono-ICI therapy is recommended following progression on platinum-based chemotherapy [6]. ICIs have substantially improved outcomes in second-line and later settings, as evidenced by large-scale randomized control trials, providing a valuable option for patients with advanced NSCLC whose disease has progressed during previous treatments [6]. Atezolizumab was approved for patients with locally advanced or metastatic NSCLC following platinum-based chemotherapy progression based on the results of the phase III OAK trial [9].

While some patients derive benefit from immune checkpoint inhibitors, others do not, and it is essential to develop better predictive factors. Potential predictive capabilities of various biomarkers have already been tested in an effort to understand this variability, but clear consensus has not yet been established [6,7].

Due to the uneven global availability of ICIs, particularly in developing nations, we found it significant to investigate the efficacy of atezolizumab as a second-line treatment option compared to its use in third-line or later settings for advanced NSCLC patients who have previously received platinum-based chemotherapy. Additionally, we wanted to identify characteristics that could be considered predictive. This study aimed to reflect the challenges faced in everyday clinical practice in different regions.

## 2. Materials and Methods

### 2.1. Patients

This retrospective study included 147 patients with advanced NSCLC that underwent treatment with atezolizumab monotherapy following disease progression on at least one prior line of platinum-based chemotherapy at two academic institutions in the Republic of Serbia. In 2019, atezolizumab monotherapy became available in Serbia for the treatment of advanced NSCLC patients whose disease previously progressed on platinum-based chemotherapy. Testing for EGFR mutations by cobas^®^ EGFR Mutation Test v2 and ALK rearrangement by immunohistochemistry was carried out in this patient population prior to initiating first line treatment. Other molecular testing was not part of the routine practice in Serbia at the time of the study’s conduction. Patients with detected driver oncogenes were excluded, so none of the included patients had a known driver oncogene. PD-L1 tumor proportion scores were tested by immunohistochemistry (Dako 22C3 clone) for patients diagnosed with advanced NSCLC after 2019. There were 31 (21%) patients with CNS metastasis prior to atezolizumab initiation. Before atezolizumab therapy was initiated, all patients received radiotherapy for CNS metastases. All included patients were adults. None of the included patients were pregnant, HIV+, or had active/chronic HBV, HCV infection, or severe renal impairment. Objective responses to atezolizumab therapy were reported according to iRECIST criteria.

### 2.2. Data

Data were retrieved from hospital-based lung cancer registries that prospectively collect demographic, clinical, pathological, and molecular characteristics as well as treatment and survival data of patients diagnosed and treated at both centers. All data were collected anonymously. This study was performed in accordance with the Declaration of Helsinki and approved by the Institutional Review Board (2268/1; 26 July 2024).

### 2.3. Statistical Analysis

Descriptive methods were used on demographic characteristics of patients. Baseline information is presented as the number of patients and percentages. Median progression free survival (PFS) and overall survival (OS) were calculated as time from the start of therapy until therapy discontinuation or date of death. Patients still alive on the last day of follow-up were censored. Median PFS and OS were estimated by the Kaplan–Meier method and compared by the log-rank test. Univariable and multivariable Cox proportional hazard regression models were used to calculate hazard ratios (HRs) and confidence intervals (CIs). In the univariate analysis, covariates included age (<70 years vs. ≥70 years), sex, histology (non-squamous vs. squamous cell lung cancer), smoking status (current vs. former or never-smoker), PD-L1 expression (≥50% vs. <50% or unknown), ECOG PS (0–1 vs. ≥2), the presence of irAEs (yes vs. no), the presence of brain metastasis at the time of atezolizumab treatment onset (yes vs. no), the use of radiotherapy (yes vs. no), as well as atezolizumab in the 2nd and/or 3rd and later lines of therapy. Multivariate analysis included variables with a significance level of *p* < 0.10 in the univariate analysis. The Chi-square test was used to determine the association between response to treatment and lines of therapy prior to atezolizumab therapy initiation. Calculated *p*-values were two-sided. We used SPSS v26 for statistical analysis.

## 3. Results

There were 147 advanced NSCLC patients that started treatment with atezolizumab who were previously treated with chemotherapy. Among them, the mean age was 60.77 (32–82), 56.5% were male, and they were mostly smokers or former smokers (53.3% and 34%, respectively). Adenocarcinomas were detected in 57.8% and squamous cell carcinomas in 38.8% of cases. Patients received 9.95 cycles of chemotherapy (1–26) on average prior to atezolizumab treatment (Table 1).

The median PFS was 7.133 months (95% CI: 4.44–9.826 months). The median OS was 38.6 months (95% CI: 33.013–44.187 months). The best overall responses to atezolizumab were complete response, partial response, and stable disease in 0.7%, 14.3%, and 42.9% of patients, respectively, adding up to an overall response rate (ORR) of 15% and a disease control rate (DCR) of 57.9%.

**Figure 1:**
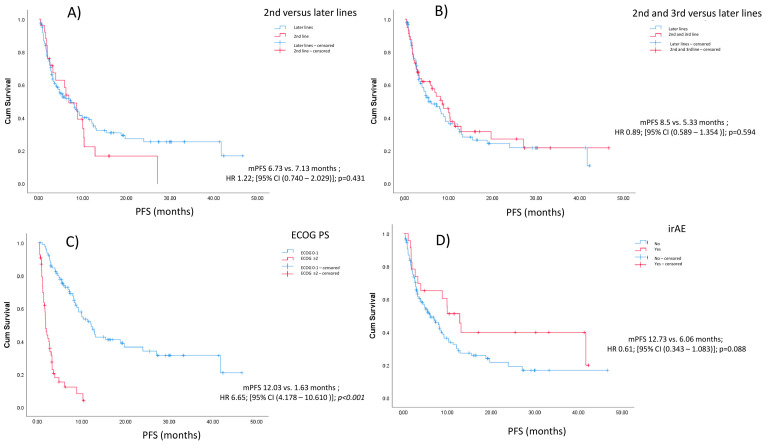
Kaplan–Meier survival curves of (**A**) patients treated in 2nd versus later lines, (**B**) patients treated in 2nd and 3rd lines versus later lines, (**C**) patients treated with ECOG 0–1 versus ECOG ≥ 2, and (**D**) patients who experienced irAEs versus those who did not.

There was no significant difference in median PFS between patients who started atezolizumab therapy in the 2nd line and those who started in later lines (6.73 vs. 7.13 months, respectively; *p* = 0.429). Similarly, there was no difference in median PFS between patients who received atezolizumab in the 2nd and 3rd lines of treatment compared to those who received it in later lines (8.5 vs. 5.33 months, respectively; *p* = 0.593) (Figure 1). The median PFS did not differ between patients who received more than 10 cycles of chemotherapy and those who received fewer than 10 cycles in previous lines (7.033 vs. 8.5 months, respectively; *p* = 0.398).

The initiation of atezolizumab therapy in the 2nd line versus later lines (*p* = 0.872) or in the 2nd and 3rd lines versus later lines of therapy (*p* = 0.398), or prior administration of fewer than 10 cycles of chemotherapy (*p* = 0.529), did not influence the overall response rate (ORR). The occurrence of immune-related adverse events (irAEs) did not significantly influence median PFS, although there was a notable numerical difference (12.733 vs. 6.067 months; *p* = 0.088) (Figure 1). There was no significant difference in median PFS when comparing non-squamous to squamous histology (7.867 vs. 7.033 months, respectively; *p* = 0.626) or when comparing PD-L1 > 50% to the rest of the patients (8.033 vs. 6.733 months, respectively; *p* = 0.739). When taking into account only patients with known PD-L1 TPS (*n* = 97; 65.9%), there was no significant difference between those with PD-L1 < 1% vs. PD-L1 ≥ 1% in terms of mPFS (4.2 vs. 5.9 months, respectively; *p* = 0.833) or mOS (27.4 vs. 30.1 months, respectively; *p* = 0.7).

Patients who experienced radiographic disease progression as the best response to chemotherapy regimens had worse median PFS on atezolizumab therapy than those who achieved at least stable disease during chemotherapy (1.63 vs. 8.16 months, respectively; *p* < 0.0001). Among patients who had progressive disease as the best evaluable response to every line of chemotherapy, there was a significantly higher prevalence of poor performance status (ECOG ≥ 2) at the time of atezolizumab therapy initiation (*p* < 0.0001).

Patients with good performance status (ECOG PS 0–1) at the time of atezolizumab initiation had significantly better median PFS compared to those with poor performance status (ECOG ≥ 2) at the time of initiation (12.03 vs. 1.633 months, respectively; *p* < 0.0001) (Figure 1). In multivariate analysis, only ECOG PS of 0–1 was predictive of prolonged median PFS (HR 6.668, 95% CI [4.173–10.655], *p* < 0.001) (Table 2).

## 4. Discussion

Real-world studies involving NSCLC patients receiving atezolizumab in the second and subsequent lines of therapy following progression on platinum-based chemotherapy are limited. Therefore, we wanted to present our experiences, which could be useful for other countries with limited access to immune checkpoint inhibitors, particularly developing nations. Our aim was to evaluate whether patients treated with atezolizumab in the second or subsequent lines benefit from ICIs and to determine if the efficacy varies when administered at different lines of treatment.

The efficacy and safety of the anti–PD-L1 immunotherapy atezolizumab in patients with previously treated advanced or metastatic NSCLC was investigated initially in a phase 2 POPLAR clinical trial, and later in a phase 3 OAK trial [10]. Primary findings from the POPLAR and OAK trials revealed significant improvements in survival outcomes, with longer OS observed in patients receiving atezolizumab versus docetaxel. These findings prompted the inclusion of atezolizumab in the second and subsequent lines of treatment in ESMO and NCCN guidelines [6,11].

In our study, the median OS was 38.6 months (95% CI: 33.013–44.187 months), which is a significant improvement over standard chemotherapy. Our results differ from those of the OAK and POPLAR studies, which had mOS rates of 13.3 and 12.6 months, respectively [12,13]. This observation may be attributed to the fact that patients in our centers had undergone a higher number of chemotherapy lines compared to those in the referenced clinical trials, thereby experiencing an extended duration of treatment prior to the administration of immune checkpoint inhibitors.

The POPLAR and OAK trials included patients who received one or two previous lines of treatment. Patients in our centers received atezolizumab in more subsequent lines—the fourth, fifth, and even sixth lines. Interestingly, there was no significant difference in the median PFS between patients who received atezolizumab in the 2nd and 3rd lines of treatment compared to those who received it in later lines (PFS 8.5 vs. 5.33 months, respectively; *p* = 0.593).

Also, the number of previously received chemotherapy cycles was irrelevant to the efficacy of ICIs in later lines. We divided patients into two groups, those who received more than ten cycles of chemotherapy and those who received less. There was no statistically significant difference in PFS between these two groups (7.033 vs. 8.5 months, respectively; *p* = 0.398).

The median PFS in our study was 7.1 months (95% CI: 4.44–9.826 months), which is higher than those in the OAK and POPLAR studies (4.2 months and 2.7 months, respectively) [12,13].

The best overall responses to atezolizumab in our study were complete response, partial response, and stable disease in 0.7%, 14.3%, and 42.9% of patients, respectively, adding up to an overall response rate (ORR) of 15% and a disease control rate (DCR) of 57.9%.

It is important to consider the patient’s performance status and clinical characteristics when planning immunotherapy, as reflected in current guidelines [6,11]. In our study, there was a clear benefit in terms of PFS for patients with good ECOG PS 0–1 compared to those with poor performance status (ECOG ≥ 2) at the time of initiation (12.03 vs. 1.633 months, respectively; *p* < 0.0001), which is concordant with previously reported results.

This suggests that immunotherapy is appropriate for patients regardless of the number of prior chemotherapy lines, provided they have a good ECOG performance status. Patients with good PS have better capability to boost their immune systems, and therefore properly respond to a cancer cell’s presence.

Few studies have addressed the issue of patients with poor PS. Registration studies for nivolumab [14,15], atezolizumab [12], and pembrolizumab [16,17,18] excluded patients with ECOG PS 2.

In Checkmate 153, patients with ECOG PS 2 had shorter OS than patients with PS 0 and 1. Patients with ECOG PS 2 had a median OS time of 4.0 months (95% CI: 3.1–6.2), with 1-year and 2-year OS rates of 24% and 9%, respectively, which was significantly lower than the median OS of the overall population (9.1 months [95% CI: 8.3–10.4]) [19]. In Checkmate 171, nivolumab had similar tolerability in patients with ECOG PS 2, but with inferior outcomes, as the reported mOS was 5.2 months (95% CI: 3.0–7.6) for patients with ECOG PS 2, as expected in this population with poor prognosis [20]. On the other hand, in the PePS2 study, the authors stated that checkpoint inhibitors can benefit patients with PS 2 with results comparable to registrational studies. The limitation of this study was the lack of a control group of patients with ECOG 0–1 [21].

Ahmed and colleagues conducted a real-world retrospective study with 285 patients with NSCLC treated with ICIs [22]. In that population, 46.3 percent had ECOG PS 2 or 3. The median OS for patients with ECOG PS 0–1 was 14.7 months, and the median OS for patients with PS 2 and 3 were 8.3 months and 1.5 months (95% CI: 1–4.9), respectively, with statistical significance (*p* < 0.0001) and similar results obtained in terms of PFS. This finding from the real-world trial is concordant with our results [22].

In the IPSOS phase 3 trial, patients who were considered ineligible to receive platinum-based chemotherapy due to either poor ECOG PS > 2, old age, or burden of comorbidities were randomized to receive single-agent chemotherapy or atezolizumab in the first line of treatment. The study found that atezolizumab improved the mOS (10.3 vs. 9.2 months; *p* = 0.028) in this patient population, making it a viable first-line treatment option for patients with advanced NSCLC who are unsuitable for platinum-based chemotherapy [23]. On the other hand, in our study, patients received atezolizumab in later lines of treatment, following disease progression on platinum-doublet chemotherapy. Additionally, platinum-based chemotherapy was used in all treatment lines preceding atezolizumab initiation. It should be noted that we conducted PD-L1 testing from initial and archival tumor samples and rebiopsies following progression. This is important to mention, as some studies have established that PD-L1 expression could be influenced by prior therapy [24]. These differences in treatment sequencing may explain the variations in outcomes observed between the two studies.

Although PD-L1 is a good predictive biomarker for first-line therapy response, it is not ideal, and according to the latest guidelines (NCCN and ESMO), combined chemotherapy and immunotherapy have their place in first-line treatment regardless of PD-L1 expression [6,11]. Mono-immunotherapy has also shown benefits in the second line regardless of PD-L1 expression. In our cohort, 66% of patients had predetermined levels of PD-L1 expression, and within this group, there was no difference in PFS and OS regardless of PD-L1 expression. This finding is consistent with the results of the OAK and POPLAR registrational studies [12,13].

Immune-related adverse events (irAEs) are associated with good therapeutic response to ICIs and prolonged PFS and OS [25,26]. In our study, only 23 patients had irAEs (15.6%) and they had greater, but not statistically significant, benefit from ICIs (mPFS 12.733 vs. 6.067 months; HR 0.61; 95% CI: 0.343–1.083; *p* = 0.088). Most irAEs were grade 1 or 2. The lack of statistical significance may be attributed to several factors, including the relatively small patient cohort and the extensive prior exposure to cytotoxic chemotherapy, which likely compromised patients’ immunological responses. Consequently, this may have diminished their ability to mount autoimmune-like reactions.

Several intriguing hypotheses have been proposed to elucidate the effects of immunotherapy when administered in later lines of treatment. Kroemer at al. state that platinum-based chemotherapy augments antitumor immunity by inducing immunogenic cell death, increasing tumor neo-antigen expression, and disturbing the immunosuppressive tumor microenvironment that prevents immune detection [27]. Chemotherapy also alters the tumor microenvironment by promoting increased infiltration of CD8 T cells, decreasing regulatory T cells and myeloid suppressor cells, and stimulating antigen-presenting cell maturation. Also, there are some clinical studies on animal models that are trying to determine how chemotherapy could enhance the effects of immunotherapy [28,29].

All these theories and evidence support the use of immunotherapy, even after initiation of treatment with standard chemotherapy protocols, if patients maintain a good clinical condition with an ECOG performance status of less than two [6,11]. This was also evident in our study.

## 5. Limitations

Atezolizumab monotherapy became available for treating NSCLC patients post-platinum-based chemotherapy progression at our center in 2019. Patients undergoing platinum-based chemotherapy in any line at that time were considered eligible to receive atezolizumab upon evidence of disease progression and the clinician’s approval. This should explain the fact that 38.1% of enrolled patients received at least three lines of platinum-based chemotherapy prior to atezolizumab. Lack of data regarding the PD-L1 status of 21% of patients could be attributed to the fact that they were diagnosed with advanced NSCLC prior to the introduction of first-line pembrolizumab therapy for the PD-L1 > 50% subset of patients, and thus PD-L1 status was not routinely tested for. As the use of atezolizumab following progression of platinum-based chemotherapy was not contingent on PD-L1 status, PD-L1 levels were not routinely tested, either from the archival sample or the one obtained post-platinum-based chemotherapy progression.

## 6. Conclusions

Our real-world retrospective study of advanced non-oncogene addicted NSCLC patients that have previously received platinum-based chemotherapy, demonstrated the benefit of atezolizumab regardless of the line of treatment. The patients with good ECOG performance status have derived the most benefit from this treatment. The benefit of atezolizumab in later lines of treatment was not dependent on factors such as sex, age, histological type, the presence of brain metastases, the development of irAEs, or PD-L1 expression. However, additional research and larger prospective clinical trials are required to confirm these findings.

## Figures and Tables

**Table 1:** Clinical and demographic characteristics of patients.

N = 147	N (%)
Mean age at treatment start (range) [years]	60.77 (32–82)
Sex	
Male	83 (56.5)
Female	64 (43.5)
Smoking status	
Current	85 (53.3)
Ex smoker	50 (34)
Non-smoker	12 (8.2)
ECOG PS	
0–1	93 (63.3)
≥2	54 (36.7)
Histological diagnosis	
Adenocarcinoma	85 (57.8)
Squamous cell carcinoma	57 (38.8)
Other (NOS)	5 (3.4)
PD-L1 status	
50–100%	13 (8.8)
1–49%	36 (24.5)
<1%	48 (32.7)
No data available	50 (34.1)
Stage at the time of diagnosis	
IIIB	31(21)
IV	116 (79)
Atezolizumab line of treatment	
II	25 (17)
III	31 (21.1)
IV	66 (44.9)
V	23 (15.6)
VI	2 (1.4)
Mean number of chemotherapy cycles (range)	9.95 (1–26)
Best response to atezolizumab (iRECIST)	
PD	(42.2)
SD	63 (42.9)
PR	21 (14.3)
CR	1 (0.7)
Real-world DCR	57.9%
Real-world ORR	15%
Median PFS (95% Confidence Interval) [months]	7.133 (4.44–9.826)
Median overall survival (95% Confidence Interval) [months]	38.6 (33.01–44.187)
Occurrence of immune-related adverse events	
Yes	23 (15.6)
No	124 (84.4)

**Table 2:** Univariable and multivariable regression analysis.

	Univariable Regression Analysis			Multivariable Regression Analysis		
	HR	95% CI	*p*	HR	95% CI	*p*
Age (< 70 years vs. ≥ 70 years)	0.893	0.521–1.530	0.680			
Sex (male vs. female)	1.162	0.772–1.748	0.473			
Histology (non-squamous vs. squamous)	0.904	0.602–1.357	0.627			
Smoking status (current vs. former or never-smoker)	1.164	0.775–1.750	0.464			
PD-L1 (≥ 50% vs. < 50% or unknown)	0.917	0.470–1.787	0.798			
ECOG PS (0–1 vs. 2)	6.658	4.178–10.610	<0.001	6.668	4.173–10.655	<0.001
2nd line vs. later lines	1.225	0.740–2.029	0.431			
2nd and 3rd lines vs. later lines	0.893	0.589–1.354	0.594			
<10 cycles of chemotherapy vs. ≥10 cycles of chemotherapy	1.192	0.782–1.795	0.400			
CNS mets	1.263	0.778–2.053	0.345			
irAE (yes vs. no)	0.610	0.343–1.083	0.092	0.624	0.350–1.113	0.110
Radiotherapy (yes vs. no)	1.138	0.729–1.778	0.569			

## Data Availability

Data presented in this study are available on request from the corresponding author.
